# Molecular Imprinting on Nanozymes for Sensing Applications

**DOI:** 10.3390/bios11050152

**Published:** 2021-05-13

**Authors:** Ana R. Cardoso, Manuela F. Frasco, Verónica Serrano, Elvira Fortunato, Maria Goreti Ferreira Sales

**Affiliations:** 1 BioMark@UC, Faculty of Sciences and Technology, University of Coimbra, 3030-790 Coimbra, Portugal; araca@isep.ipp.pt (A.R.C.); mffrasco@uc.pt (M.F.F.); vmserrano@uc.pt (V.S.); 2 BioMark@ISEP, School of Engineering, Polytechnic Institute of Porto, 4249-015 Porto, Portugal; 3 CEB, Centre of Biological Engineering, University of Minho, 4710-057 Braga, Portugal; 4 i3N/CENIMAT, Department of Materials Science, Faculty of Sciences and Technology, Universidade NOVA de Lisboa and CEMOP/UNINOVA, 2829-516 Caparica, Portugal; emf@fct.unl.pt

**Keywords:** molecular imprinting technology, nanozymes, enzyme-like activity, biosensing, biomimetics

## Abstract

As part of the biomimetic enzyme field, nanomaterial-based artificial enzymes, or nanozymes, have been recognized as highly stable and low-cost alternatives to their natural counterparts. The discovery of enzyme-like activities in nanomaterials triggered a broad range of designs with various composition, size, and shape. An overview of the properties of nanozymes is given, including some examples of enzyme mimics for multiple biosensing approaches. The limitations of nanozymes regarding lack of selectivity and low catalytic efficiency may be surpassed by their easy surface modification, and it is possible to tune specific properties. From this perspective, molecularly imprinted polymers have been successfully combined with nanozymes as biomimetic receptors conferring selectivity and improving catalytic performance. Compelling works on constructing imprinted polymer layers on nanozymes to achieve enhanced catalytic efficiency and selective recognition, requisites for broad implementation in biosensing devices, are reviewed. Multimodal biomimetic enzyme-like biosensing platforms can offer additional advantages concerning responsiveness to different microenvironments and external stimuli. Ultimately, progress in biomimetic imprinted nanozymes may open new horizons in a wide range of biosensing applications.

## 1. Introduction

Enzymes are unique natural catalysts with outstanding efficiency and substrate specificity. Their use in bioanalytical methods, e.g., glucose oxidase (GOx) for glucose detection and horseradish peroxidase (HRP) for enzyme-linked immunosorbent assay (ELISA), is extensive, as it has always attracted research due to their tremendous potential [1]. However, natural enzymes have limiting features linked to easy denaturation, and limited temperature and pH ranges for optimal activity, hampering many of the foreseen applications [1,2]. The disadvantages related to poor stability and reusability, along with high costs for preparation and purification, have led to efforts in designing synthetic mimics [3]. The research in this field spreads from semisynthetic approaches (e.g., genetic modification of natural enzymes) to artificial systems (e.g., cyclodextrins, metal complexes, porphyrins, dendrimers, polymers) [2,4]. These biomimetic materials are characterized by unique features and have several advantages compared with natural enzymes. The artificial enzymes are low-cost, have easy mass production, high stability (especially at high temperature), and long-term storage feasibility [4]. The progress in the field of nanomaterial-based artificial enzymes or nanozymes has been fast since the first discovery of the unexpected peroxidase-like activity of ferromagnetic nanoparticles [5]. Thus, nanozymes are considered alternatives to natural enzymes and the prospective biomedical applications are vast, ranging from disease diagnostic and imaging to therapeutics [6,7]. Considering biosensing devices and the current technological advances, synergistic effects are expected to achieve ultrasensitive methods, such as colorimetric, fluorometric, chemiluminescent, surface-enhanced Raman scattering, and electrochemical [7,8], with special emphasis on the electrochemical-based devices, which offer great advantages in terms of portability and feasibility of point-of-care use. Despite these enthusiastic perspectives, poor substrate selectivity, low efficiency and limited catalytic types are challenges to be tackled. In a biomimetic convergence, molecularly imprinted polymers (MIPs) are synthetic highly selective receptors, which have been integrated with nanozymes to improve the desired features, with special outcomes for sensor design. The current review starts by providing an overview on the composition, enzyme-like activities for signal production, and ability to tune the properties of nanozymes. It is followed by addressing the bases of MIP technology, fabrication methods, and function as synthetic catalysts. Finally, emphasis is given to the most recent progress on creating MIPs on nanozymes along with detection systems, and the future perspectives of this exciting field of research are summed up to conclude.

## 2. Design of nanozymes

As natural enzymes are efficient biocatalysts, there has always been great interest in mimicking the proven high substrate specificity and superior catalytic activities. Moreover, the development of synthetic approaches could overcome the disadvantages of natural enzymes related to high-cost preparation and purification, low stability, and difficulties in storage and reuse [1,3]. Thus, allied with advances in nano- and biotechnology, artificial enzyme mimics have been extensively studied [7,9]. 

**Figure 1:**
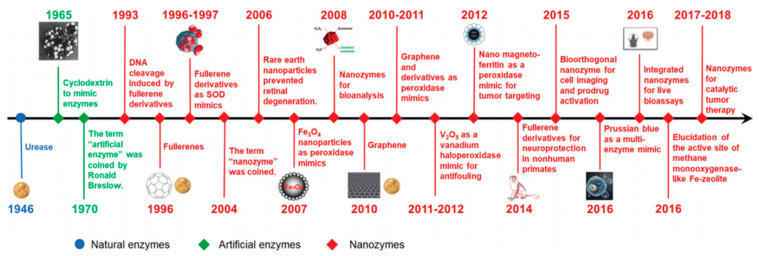
Timeline highlighting relevant historical benchmarks of nanozyme development. (Reproduced with permission [9], Copyright 2019, The Royal Society of Chemistry).

The term “nanozyme” appeared for the first time in a work by Scrimin, Pasquato, and co-workers in 2004 to describe the excellent catalytic properties of a multivalent system comprising ligand-functionalized thiols on Au nanoparticles (NPs) [10]. Since the notable discovery of the intrinsic catalytic activity of magnetic Fe_3_O_4_ NPs as peroxidase in 2007, nanozymes refer to nanomaterials with enzyme-like characteristics [5,9]. The field has been expanding quickly and nanomaterials with enzyme-mimicking activities, like Au NPs, Fe_3_O_4_ NPs, fullerene derivatives, among many others, have attracted great interest and led to important benchmarks [9,11] (Figure 1).

**Figure 2:**
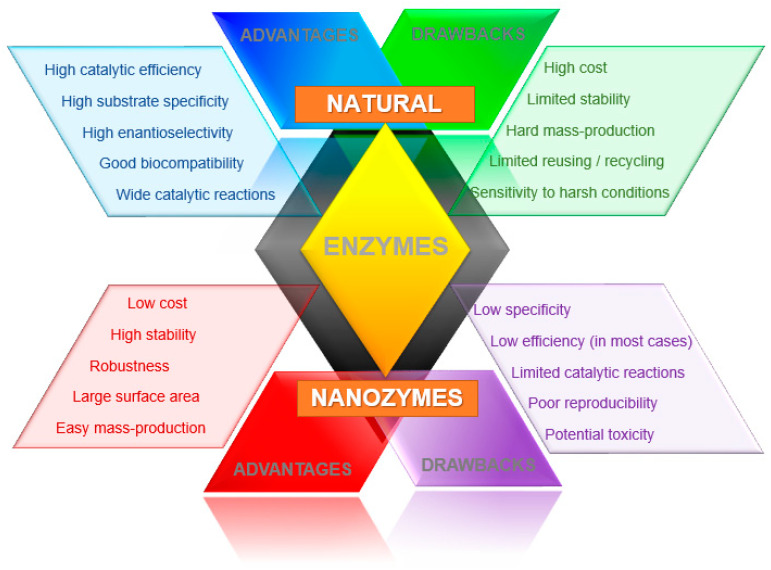
Comparison of some characteristics between nanozymes and natural enzymes.

Nanomaterials as enzyme mimics present many advantages in comparison with natural enzymes. Either as single or multi-components like composites or doped materials, nanozymes have high surface-to-volume ratio, tunable catalytic activity, and plenty of surface reactive species [4,12]. These features are combined with cheaper and simpler manufacturing processes, long-term stability, and robustness to harsh environments. However, some other traits are not so favorable, such as toxicity in biological systems and lack of selectivity, and the catalytic activities of most nanozymes is still low, which may limit the range of applications. Thus, nanozymes as artificial catalysts are a likely choice, but the field still faces many challenges (Figure 2).

### 2.1. Classification and Enzyme-Like Activities

**Figure 3:**
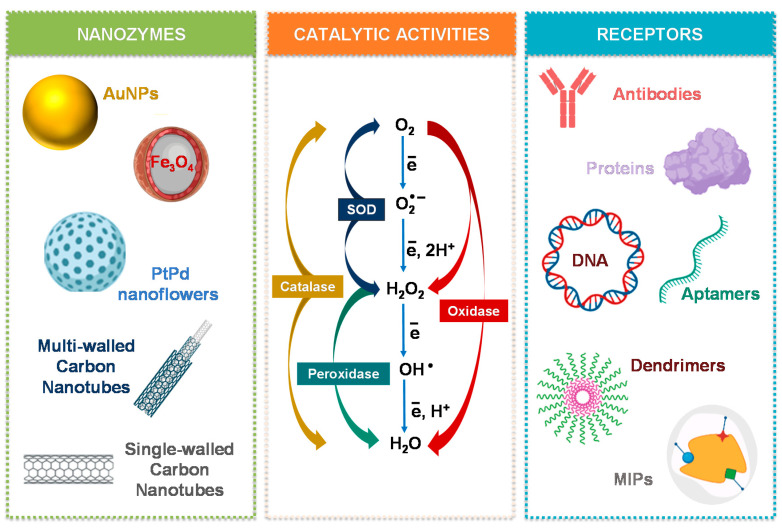
Schematic illustration of some nanomaterials mimicking enzymes, the most studied enzyme-like activities, and common ligands to tune the catalytic efficiency and selectivity of nanozymes.

Regarding artificial enzyme mimics, several nanomaterials with inherent catalytic activity have been studied as highly stable and low-cost approaches. These nanomaterials can be categorized in main groups regarding their composition, namely into metal, metal oxide, carbon-based materials, and their hybrids [13]. A variety of nanomaterials has been studied as artificial enzyme mimics, including CeO_2_ NPs, Fe_3_O_4_ NPs, Pt nanomaterials, Au NPs, bimetal and trimetal NPs, graphene oxide, carbon nanotubes, fullerene derivates, quantum dots, metal organic frameworks (MOFs) as some common examples [3,7,14,15,16] (Figure 3).

The discovered nanozymes so far can broadly function as oxidoreductases, namely as peroxidase, oxidase, superoxide dismutase (SOD), and catalase mimics [9]. Other enzyme-like activities by carbon-based materials, Zr- and Cu-based MOFs and Au NPs modified with catalytic monolayers have also been reported, namely, hydrolase activity, which catalyzes chemical bond hydrolysis (e.g., nuclease, esterase, phosphatase, protease, and silicatein) [7,9]. Laccase-like activity has been demonstrated in Cu-based MOFs based on guanosine monophosphate coordinated copper [17]. Multi-nanozymes where two or more types of enzymes are mimicked can also be observed in some nanomaterials like Au, Pt, and CeO_2_ [9,18,19]. These capabilities can be expanded by using bimetallic NPs that can achieve not only single- but also multiple-enzyme mimicking, or nanocomposites to benefit from their synergistic effects and enhanced catalytic performance [20,21] (Figure 3). 

**Figure 4:**
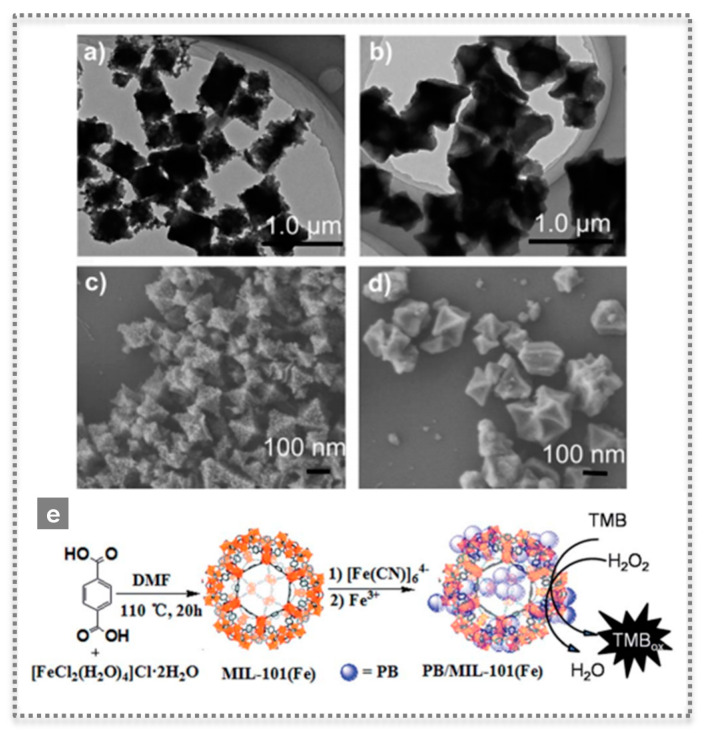
Characterization by TEM (**a**,**b**) and SEM (**c**,**d**) of microporous MOF MIL-101(Fe) as-prepared (**a**,**c**) and after introducing Prussian blue, PB/MIL-101(Fe) (**b**,**d**), as highly efficient peroxidase-like mimics, and scheme illustrating the synthesis of PB/MIL-101(Fe) (**e**) (Reproduced with permission [23] Copyright 2015, The Royal Society of Chemistry).

**Figure 5:**
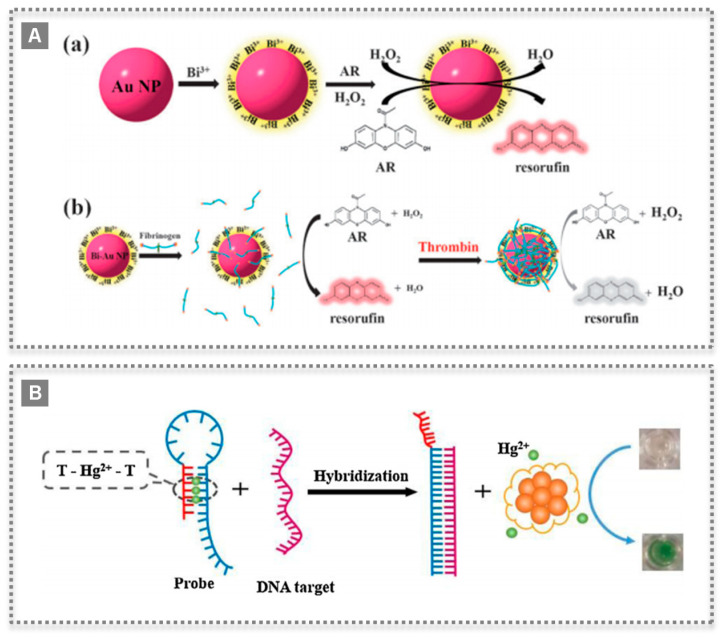
Nanozyme bioconjugates for sensing applications: (**A**) Scheme of bismuth-gold nanoparticles preparation and peroxidase-like catalyzing mechanism for reaction with Amplex Red (AR) (**a**), and further modification with fibrinogen as probe for detection of thrombin (**b**). (Reproduced with permission [25] Copyright 2012, The Royal Society of Chemistry); (**B**) Colorimetric biosensor based on oxidase-like activity of bovine serum albumin-protected silver clusters, which is switched on selectively by mercury ions and applied to detect DNA. (Reproduced with permission [30], Copyright 2015, Elsevier B.V.)

Peroxidases, such as HRP, are widely used in biosensor devices and they catalyze the oxidation of substrates by a peroxide, such as H_2_O_2_. After finding the novel properties of Fe_3_O_4_ magnetic NPs, their peroxidase-like activity was used in the detection of H_2_O_2_ and glucose [22]. The nanozyme catalyzed the oxidation of the substrate 2,2′-azino-bis(3-ethylbenzothiazoline-6-sulfonic acid)-diammonium salt (ABTS) by H_2_O_2_ to the oxidized colored product. Moreover, by combining this reaction with the catalytic oxidation of glucose by using GOx, it was possible to develop a colorimetric assay for glucose detection [22]. Since then, the field has evolved rapidly and metals, metal oxides, MOFs and carbon-based nanomaterials have been studied as peroxidase-like mimics [23,24]. In a work by Cui et al., 2015, a simple colorimetric biosensing platform was proposed based on growing Prussian blue (PB) on the microporous MOF MIL-101(Fe), forming uniform octahedral nanostructures with highly efficient catalytic activity for H_2_O_2_ [23]. PB crystals are made of iron ions coordinated by CN bridges, possess a high surface area, also exhibiting intrinsic high peroxidase-like activity. Moreover, the outer surfaces of these PB/MIL-101(Fe) nanostructures were successfully modified, rendering them biocompatible while maintaining their activity [23] (Figure 4). Another interesting example was the application of a peroxidase-like activity to successfully detect thrombin in plasma by preparing a fibrinogen-modified bismuth-gold (Fib-Bi-Au) NPs [25]. The Fib-Bi-Au NPs catalyzed the oxidation of Amplex Red in the presence of H_2_O_2_, and this simple fluorescence-based assay enabled detecting thrombin with limit of detection of 2.5 pmol L^−1^ and revealing a promising clinical application [25] (Figure 5A). The majority of nanozymes applied in detection exhibit peroxidase-like activity; thus, numerous examples are found in the literature with a broad range of nanomaterials [7,26]. Recently, a covalent organic framework (COF) nanozyme has been developed by incorporating an iron porphyrin unit in the COF backbone as the active center and L-histidine as the substrate binding site for selective chiral recognition. The peroxidase-like activity was shown to be enantioselective and with higher activity than the natural HRP, while both the activity and selectivity can be easily modulated by changing the doped amino acids and their content [27]. 

**Figure 6:**
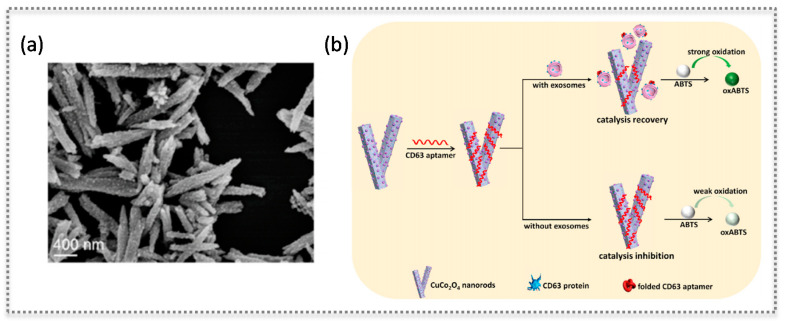
Nanozyme based on CuCo_2_O_4_ nanorods: SEM characterization (**a**) and scheme showing the modulation of oxidase-like activity of the nanorods by the adsorption of the aptamer that recognizes CD63 protein on exosomes (**b**), as a label-free sensing approach. (Reproduced with permission [31], Copyright 2020, Elsevier B.V.).

Oxidase-like nanozymes are those that catalyze the oxidation of substrates with molecular oxygen. Natural enzymes are usually selective for a given substrate, hence their names such as GOx. Nanomaterials such as Au NPs, MnO_2_, or CeO_2_ exhibit oxidase-like activity, but they lack selectivity to a given substrate [9,28,29]. Still, oxidase-like nanozymes are integrated in sensors with proper coupling to biomolecules pertaining to the target. For instance, a colorimetric sensor for mercury ions and DNA molecules relied on the oxidase-like activity of bovine serum albumin-protected silver clusters (BSA-Ag NCs). This activity was stimulated by mercury showing high catalytic activity towards 3,3′,5,5′-tetramethylbenzidine (TMB), in a “switched-on” state [30]. Moreover, as mercury ions are known to bind with two DNA thymine bases, forming base pairs, the sensor could detect DNA molecules. A hairpin structure containing mercury ions was disrupted in the presence of the target DNA, releasing the ions that switched on the oxidase mimicking activity of BSA-Ag NCs. The target DNA could be detected as low as 10 nmol L^−1^, with a linear range from 30 to 225 nmol L^−1^ [30] (Figure 5B). In a recent work, the oxidase-like activity of Cu/Co bimetallic MOF functionalized with an aptamer was used to detect a protein on the surface of exosomes [31]. The prepared CuCo_2_O_4_ nanorods were surface-modified with CD63 aptamers resulting in catalysis inhibition. The aptamers disassembled upon exosome recognition originating a recovery of the oxidase-like activity. This colorimetric method enabled the detection of exosomes in a range of 5.6 × 10^4^ to 8.9 × 10^5^ particles µL^−1^, with a detection limit of 4.5 × 10^3^ particles µL^−1^ [31] (Figure 6).

Both SOD and catalase enzymes are involved in protecting cells from oxidative damage, and likewise to other enzyme mimics, CeO_2_, Au NPs, among other nanozymes, are artificial alternatives with many potential therapeutic applications [13,32]. Because of their antioxidant properties, many works study the potential benefits of nanozymes with catalase and SOD activities for cell and tissue protection against oxidative stress, in cancer therapy, and as anti-inflammatory and antibacterial agents [33,34]. 

### 2.2. Tuning Nanozymes Properties

The catalytic activity of nanozymes is related to the physicochemical properties of the nanomaterials, mainly by their atomic composition, i.e., by atoms present both in the inside core and on the surface [34]. Other factors that affect the enzymatic activity include size, morphology, surface coating and modification, pH, and temperature [35,36]. As a result of their large surface area that is easily modified by bioconjugation, it is possible to tune some properties like size, shape, and composition, leading to final materials with improved (or distinct) catalytic activities, robustness to changes in the microenvironment, and high stability [8,37]. The fact that some nanomaterials also possess innate magnetic and optical properties, like Fe_3_O_4_ and Au NPs, is seen as an additional gain to obtain multiple functionalities that are highly valuable for bioaffinity separation or biosensing applications [38]. 

One of the major drawbacks of nanozymes is the lack of selective recognition of the substrate in contrast to the corresponding natural enzymes. Thus, various methods have been pursued to improve the selectivity of nanozymes. One approach has been to couple peroxidase-like nanozymes with oxidases, producing H_2_O_2_ only in the presence of specific substrates [39]. Surface modification with proper ligands or bioreceptors as antibodies, aptamers, and oligonucleotides, through bioconjugation or physisorption, has also been attempted [1] (Figure 3). In this context, the combination of nanozymes with MIPs has opened a new avenue to overcome this technological challenge [36]. The area of biomimetic catalysis is vast, and readers are referred to comprehensive literature reviews focusing on types of nanomaterials, classification, catalytic mechanisms, activity, and applications of nanozymes [6,7,9,11,33].

## 3. Molecular Imprinting Technology

Over the past decades, MIPs have proven excellent features in mimicking natural recognition events for a myriad of applications. The most compelling examples concern the replacement of natural antibodies in diagnostic-based immunoassays [40,41]. The molecular imprinting technology has matured to the point of reaching similar or even surpassing the required characteristics in terms of selectivity, robustness, and cost-effective production without involving animal research [42]. Thus, the combination of “plastic” molecular recognition with advances in sensor fabrication has been offering selective and sensitive methods. These devices are promising for low-cost and rapid detection and monitoring of biomarkers of disease (e.g., protein, nucleic acids, metabolites, virus) and compounds of interest in environmental and food analysis [43]. Besides sensors [44], MIPs have several applications, e.g., in drug delivery, tissue engineering, biocatalysis, bioimaging, extraction, among others [45,46,47]. Given the known advantages of these synthetic materials, research on building catalytic activity into MIPs has for long been considered an exciting area [40]. 

### 3.1. MIPs Design

**Figure 7:**
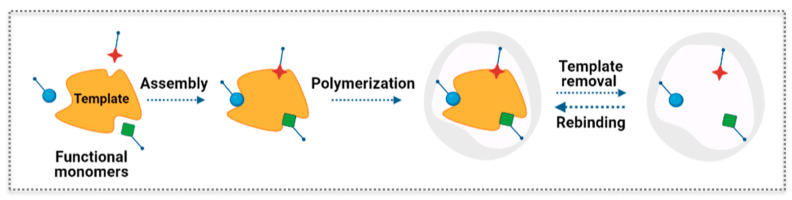
Scheme of the principle of molecular imprinting recognition.

The general concept behind the molecular imprinting technology is to build a synthetic polymer receptor via template-guided synthesis. In general, the typical steps for the synthesis of MIP materials can be described as schematically represented in Figure 7. This includes the following steps: (1) the template or print molecule is mixed with functional monomers that get positioned spatially around the template and guide the assembly; (2) the polymerization reaction creates a crosslinked matrix around the template; (3) the template is removed from the polymer network leaving a complementary cavity that retains the spatial features, i.e., size and shape, and bonding preferences of the template; (4) the imprinted polymer selectively rebinds the template from complex samples [48]. Different interactions can be established between the template and functional monomers, namely, noncovalent (e.g., hydrogen bonds, ionic interactions, and hydrophobic effects), covalent, and semi-covalent bonding [42,48,49]. Owing to its versatility, the noncovalent approach is the most widely applied method. The best conditions for MIP synthesis can be studied by computational modelling and combinatorial methods [50,51]. Several parameters are relevant to verify the recognition properties of the MIP and its successful application, namely in sensors. Those include the adsorption capacity, imprinting factor, selectivity factor, and response time [52,53].

Concerning the polymerization methods, different synthetic routes can be used for MIP preparation. The most widespread are free radical polymerization [54] and sol–gel process [55]. In free radical polymerization, it is possible to select among bulk polymerization (that includes mechanical grinding and sieving) and more complex techniques, for example, to originate particles (e.g., suspension, precipitation, emulsion, and seed polymerizations), which are also commonly used [56]. When MIPs are combined with electrochemical detection, electropolymerization is the preferred approach [44], with main advantages relating to direct formation of the polymer film on the surface of the transducer in a wide range of substrates [57,58,59] allowing the production of both conductive and nonconductive polymers [60]. Besides the mentioned methods for MIP synthesis, it is also possible to use other strategies for its production, such as surface imprinting, living polymerization, solid-phase MIP nanoparticles, and various novel technologies that have emerged [49,61]. The methods chosen for MIP production with desirable properties and the final configurations clearly depend on the target molecule (e.g., the whole target or a small fragment as an epitope, small, or large macromolecules) and application, also considering the costs and simplicity of the processes. MIPs is a thriving research subject and comprehensive reviews can be found in the literature covering methods of polymer fabrication and imprinting strategies, range of applications, and integration in biosensing devices [43,46,56,62,63].

### 3.2. MIPs as Biomimetic Catalysts

The first examples of imprinted polymers as artificial enzymes were reported in the late 1980s [2,41]. The ability to tailor the imprinted sites with functional groups, allied with the robustness and stability of polymer materials, led to considerable research efforts to expand the catalytic applications of MIPs. Many different approaches have been explored (e.g., (non)covalent imprinting, chemical reactions) and catalytic MIPs have been prepared using analogues of substrates, transition states, or products as templates [64,65,66,67,68]. Since the first important developments, considerable advances in the field have allowed to prepare more flexible and adaptative structures [2,40].

Imprinted polymers have been combined with amino acids, peptides, metals, among others, in synergic strategies to realize the potential of artificial enzymes [69,70,71]. For example, peroxidase mimics are useful in the detection of the relevant biomarker for tumor diagnostic 5-hydroxyindole-3-acetic acid (5-HIAA), which is an indoleamine metabolite and is simultaneously a substrate for peroxidase activity [72]. To this end, a multifunctional MIP material was prepared as an artificial peroxidase enzyme. The MIP with hemin as catalytic center and 5-HIAA as template was obtained by fabricating a core-shell magnetic MIP and demonstrated selective oxidation of 5-HIAA. Then, the products of this oxidation were separated and detected by high-performance liquid chromatography (HPLC) to obtain a quantitative detection of 5-HIAA [72]. 

In biomimetic catalysis, peptide-based enzyme mimicking is also of interest. Major advantages are related to the fact that the building blocks of peptides are amino acids that work as catalytic groups, assemble, and form supramolecular structures through noncovalent interactions, all traits of natural enzymes [73]. Based on this approach, many artificial enzymes have been constructed, such as hydrolases, aldolase, oxidoreductase, among others [73,74,75]. As for other synthetic alternatives, lower catalytic activity and substrate specificity are still challenging, and molecular imprinting can contribute to improving those features. By combining MIPs with peptide assemblies, interesting peptide-based artificial enzymes have been developed. The peptide Fmoc-Phe-Phe-His (Fmoc-FFH) forms stable nanofibers through self-assembly and has the catalytic activity of histidine [75]. Thus, when an MIP for the substrate *p*-nitrophenyl acetate is prepared on the surface of the catalytic nanofibers, specific binding sites are provided for enhanced artificial hydrolase activity. In addition, the catalytic ability was improved in a wider reaction temperature and pH, and the catalyst was more easily recyclable owing to the introduction of the polymer layer [75]. Another example is the work of Li et al., 2020, who developed a peroxidase-like enzyme by the co-assembly of Hemin and Fmoc-FFH, combined with an imprinted polymer of the substrate ABTS. Since the polymer components can be easily adjusted, the addition of a cationic monomer further enhanced the catalytic activity because the electrostatic interaction created a synergistic effect [73]. 

DNA oligonucleotides can also have functional properties, such as in molecular recognition and catalysis, the latter known as DNAzymes [76]. Many peroxidase mimics are DNA-based catalysts and oxidize common substrates like TMB and ABTS in the presence of H_2_O_2_, which is of interest because colorimetric products enable the development of optical sensors [77]. To improve the selectivity of a peroxidase-mimicking DNAzyme based on a guanine-quadruplex (G4) DNA with a hemin cofactor, an MIP layer for proper substrates was prepared. The DNAzyme incorporated into the MIP exhibited expected selectivity, as well as enhanced stability (DNA degradation was reduced) and activity (higher than that of free DNAzyme) [77,78]. A very promising application of these imprinted DNAzyme nanogels is for biocatalysis inside cells and intracellular therapeutic applications. This material was efficiently internalized, and the MIP was more effective inside cells than the nonimprinted control, leading to the conclusion that the imprinted matrix was effective when located intracellularly [78].

**Figure 8:**
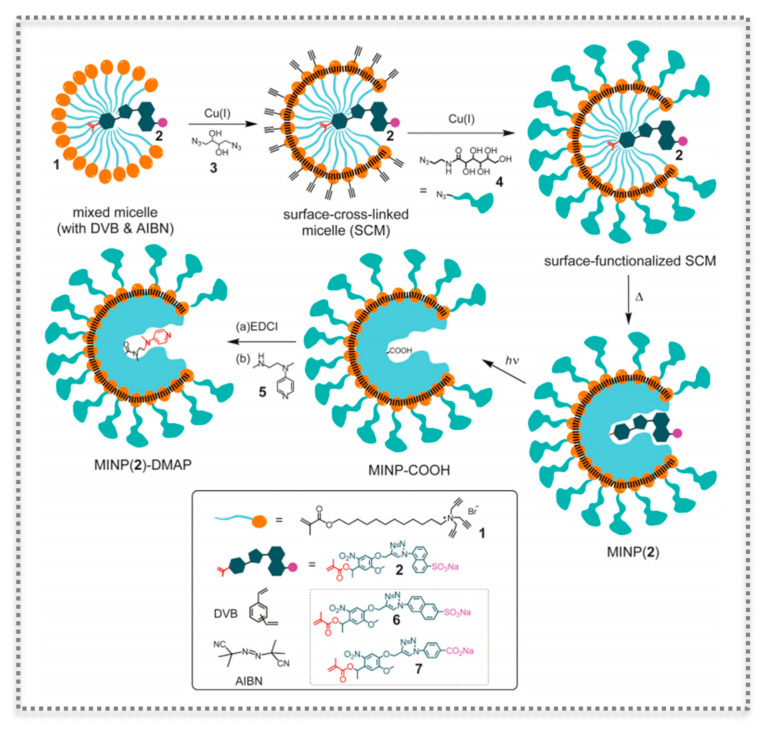
Preparation of catalytic molecularly imprinted nanoparticles (MINPs) functionalized with the transacylation catalyst 4-dimethylaminopyridine (DMAP), as a method to molecularly imprint within cross-linked micelles. (Reproduced with permission [79], Copyright 2017, Wiley-VHCA AG).

Molecular imprinting technology is extremely versatile as demonstrated by the possibility of imprinting within the nanospace of doubly cross-linked micelles [79,80] (Figure 8). In this method, the imprinting is confined within the boundary of surfactant micelles. The obtained MIP NPs have a hydrophobic/hydrophilic core-shell morphology and are water-soluble, making this micellar imprinting very useful for a variety of template molecules [79]. Such imprinted NPs can also be post-functionalized to present catalytic properties. In addition, the catalysis can be fine-tuned owing to the facile modification of size, shape, and depth of the binding pockets. The technology was demonstrated to deploy very efficient artificial phosphodiesterase and esterase-like activities [79,80].

## 4. Nanozymes@MIPs

Recent advances on tailored MIPs on nanozymes represent a novel avenue for additional advances in the field. The synergistic catalysis arising from integrating two different artificial enzymes was first advanced as a strategy for the formation of disulfide bonds in peptides [81]. In this study, imprinted polymeric microzymes and inorganic (Fe_3_O_4_) nanozymes were integrated in one process resulting in high product yields and excellent selectivity [81].

Further works expanded the concept based on coating the nanozymes with the MIP layer containing binding pockets to improve the selectivity and the catalytic performance of nanozymes [82]. Generally, the nanozymes do not present selective recognition, and a specific bioligand (e.g., antibody, aptamer) is conjugated on the nanozyme. The use of such ligands may compromise the high stability and low cost of nanozymes. Furthermore, the natural ligands available may not cover the range of emerging analytes [82,83]. These drawbacks can be addressed by growing artificial substrate recognition sites on the nanozymes by molecular imprinting technology. The MIP layer provides the selectivity, but it has been reported that the catalytic activity of various nanozymes is also enhanced. Considering nanozymes as heterogenous catalysts, the substrate must diffuse to the catalyst surface, and after the reaction, the product desorbs, enabling enzyme regeneration [84]. Interestingly, if the MIP is grown on the nanozyme, the several steps of catalysis could be enhanced or inhibited by the polymer layer. Thus, a surface science approach supported the study of the three reaction steps to explain the enhanced catalytic activity in the presence of the MIP. This work suggested that the substrate concentration near the nanozyme surface was enriched by the imprinted polymer with faster transportation kinetics. Moreover, the activation energy was lower, and the MIP did not retain the products, facilitating enzyme turnover [84].

**Figure 9:**
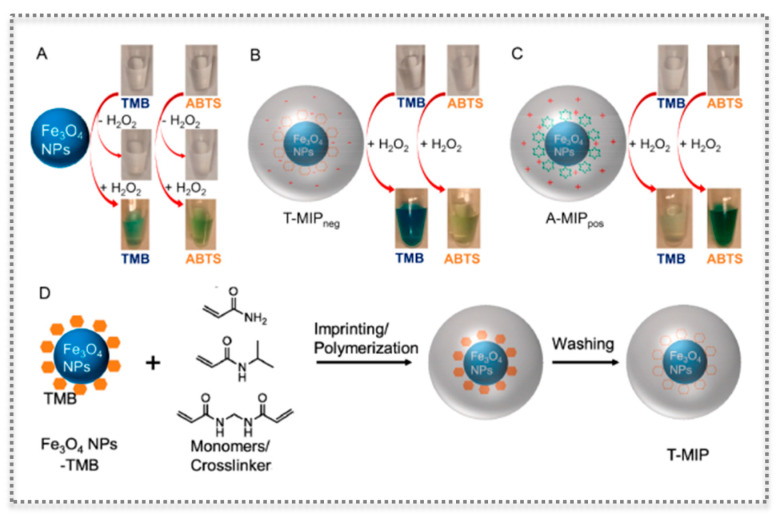
Molecular imprinting on nanozymes: Photographs and schemes showing bare Fe_3_O_4_ nanozyme (**A**), with an anionic MIP layer (T-MIP_neg_) (**B**) and with a cationic MIP layer (A-MIP_pos_) (**C**) for oxidizing two substrates (TMB and ABTS), as well as scheme of imprinting the nanogel (**D**). (Reproduced with permission [82], Copyright 2017, American Chemical Society).

For most nanozymes@MIPs, the imprinted polymer is grown to entrap the nanozyme. An example of such strategy has been presented by creating substrate binding cavities on three classic nanozymes with peroxidase- and oxidase-like activities [82] (Figure 9). The imprinted nanogels, prepared by aqueous precipitation polymerization, were synthesized on Fe_3_O_4_ nanoparticles with peroxidase-like activity, but also on CeO_2_ mimicking oxidase activity and Au NPs mimicking peroxidases [82]. The substrate binding pockets enabled achieving remarkable specificity and the enhancement of the activity of nanozymes. In the case of Fe_3_O_4_ and CeO_2_, two substrates were imprinted, namely, TMB and ABTS, that when oxidized show a blue and a green color, respectively. The imprinted substrates for Au NPs were TMB and dopamine [82]. The prepared MIP appeared to be porous, allowing an efficient substrate diffusion. Interestingly, and in accordance with some reports in the literature, surface modification of nanozymes can enhance their activity [82]. The MIPs, which were properly engineered with functional charged monomers, highly enhanced the selective substrate recognition and the catalytic activity. The improvement caused by the MIP was consistent among the various nanozymes and substrates analyzed, suggesting that nanozyme@MIP may be a general method to obtain desired selectivity and activity [82].

Other examples in the literature can be found, such as growing a polypyrrole (PPy) based MIP on Fe_3_O_4_ nanozymes using methylene blue (MB) as substrate [85]. In this study, the Fe_3_O_4_@PPy composite presented superior catalytic properties for MB in the presence of sodium persulfate, a sulfate radical-based oxidant, in comparison with bare Fe_3_O_4_ nanozymes. Interestingly, Fe_3_O_4_@PPy could still degrade more than 80% of MB after five recycling cycles [85].

The versatility of imprinted nanozymes was tested when trying to develop a universal sensor for multiplex detection. This study used peroxidase-like metal (Pt, Ru, and Ir) nanozymes to fabricate cross-reactive sensor arrays to detect a variety of analytes [83]. The sensor arrays allowed to discriminate several small biothiol molecules, proteins, and cells. Other successful traits were related to the ability of identifying unknown samples, as well as discriminating biothiols in serum and proteins in human urine [83].

The use of Au NPs in the construction of imprinted nanozymes, as a novel method for enhanced selective detection of glucose, was conceived by employing aminophenylboronic acid (APBA) in the MIP shell [86]. The affinity to glucose is assured by the boronic group in APBA, which can bind to adjacent hydroxyls of saccharides under alkaline conditions. Moreover, to improve the catalytic activity, heptadecafluoro-*n*-octyl bromide nanoemulsion was introduced to provide oxygen, which resulted in efficiency gain of about 270-fold [86]. These Au NP-based GOx mimics are very promising considering the importance of glucose monitoring.

The oxidase-like activities of other metal nanoparticles have been explored, such as the case of Au–Pt alloy [87]. This alloy was coupled to magnetic microspheres to enhance the stability and ensure their magnetic separation. The affinity to substrate was accomplished by preparing a MIP containing APBA as polymer shells with imprinted sites for glucose. Having a GOx-like activity, the imprinted nanozymes had about 200-fold higher catalytic efficiency than Au NPs [87].

**Figure 10:**
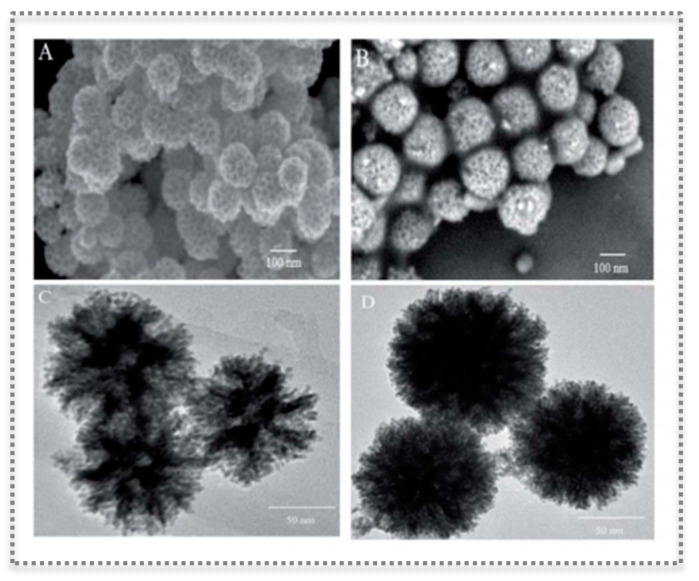
Characterization of molecular imprinting on PtPd NFs showing spherical nanostructure with flower-like morphology and mesoporous on the surface: SEM images of PtPdNFs (**A**) and T-MIP-PtPd NFs (**B**); TEM imagens of PtPd NFs (**C**) and T-MIP-PtPd NFs (**D**). (Reproduced under the terms and conditions of the Creative Commons Attribution Non-Commercial Unported 3.0 License [88], Copyright 2019, published by The Royal Society of Chemistry).

Recently, PtPd nanoflowers (NFs) exhibiting peroxidase-like activity were synthesized by a surfactant-directing method and further surface-modified with a MIP layer [88]. The MIP was prepared by aqueous precipitation polymerization and contained imprinted pockets for TMB (Figure 10). The final composite (T-MIP-PtPd NFs) had better catalytic properties, achieving a linear range of 0.01–5000 µmol L^−1^ and a detection limit of 0.005 µmol L^−1^ for colorimetric detection of H_2_O_2_. A sensitive colorimetric detection of glucose was also possible through a cascade reaction employing GOx [88].

The research on nanocomposites to gain from a synergistic effect has been investigated using a combination of PtCu bimetallic NPs and poly(styrene sulfonate) (PSS) functionalized graphene (Gr). Moreover, the surface of PtCu/PSS-Gr was covered by a MIP for detection of the flavonoid puerarin. The peroxidase-like activity of the MIP@PtCu/PSS-Gr was applied in the colorimetric detection of puerarin reaching a limit of detection of 1 × 10^−5^ mol L^−1^ and with a linear range of 2 × 10^−5^ to 6 × 10^−4^ mol L^−1^ [89]. As proposed by this study, the PSS-Gr had good dispersity, stability, and a large surface area, which supported the dispersion of PtCu NPs, i.e., preventing any possible agglomeration that could lead to reduced enzymatic activity. Additionally, as the PtCu/PSS-Gr nanocomposite was covered by the MIP, without the target analyte puerarin, the small H_2_O_2_ passes through the polymer and reaches the peroxidase-like enzyme, generating hydroxyl radicals that trigger the oxidation of TMB. However, when puerarin is present and specifically binds the imprinted sites, it acts as barrier to H_2_O_2_, leading to decreased catalytic reaction [89].

Photooxidase mimics are activated by light for the oxidation of the substrate in the presence of dissolved oxygen [90]. Among the variety of nanomaterials that have been used as photooxidase mimics, the graphite carbon nitride (g-C_3_N_4_) is an emerging visible-light-active organic semiconductor with intrinsic fluorescence properties. Hence, applications of g-C_3_N_4_ in photocatalysis and photoluminescence-based biosensing are growing [90]. A recent study demonstrated the interesting properties of surface molecular imprinting on g-C_3_N_4_ nanozymes for improved detection of L-cysteine in serum [90]. The enzymatic activity was first probed upon blue LED irradiation, leading to oxidation of the chromogenic substrate like TMB without destructive H_2_O_2_. Most interestingly, the MIP-g-C_3_N_4_ nanozyme, i.e., having TMB imprinted sites on the surface of g-C_3_N_4_, showed to suppress the matrix interference from serum samples, enhancing both substrate selectivity and enzyme activity in comparison to bare g-C_3_N_4_. Superior properties in terms of enzyme affinity to TMB, in comparison to other inorganic nanozymes, were also suggested in this study [90]. 

Electrochemiluminescence (ECL) detection has been highly investigated in sensor development. Nonetheless, common ECL reagents have a few disadvantages (e.g., high toxicity, low stability, and environment-sensitive luminescence efficiencies) and aggregation-induced emission (AIE) materials can overcome these limitations in some practical applications [91]. Additionally, nanozyme amplification has been proposed to offer unique advantages to ECL, and an aggregation-induced (AI)-ECL assay combined with Co_3_O_4_ nanozymes has been developed for the detection of antibiotic residues, namely, chloramphenicol [91]. In this sensor, the strong and stable signal relied on COF materials with AI-ECL groups (COF-AI-ECL), while the Co_3_O_4_ nanozymes worked as the amplification element. The synthesized COF-AI-ECL and Co_3_O_4_ were cross-linked to the surface of a gold electrode, followed by the construction of a MIP for selective recognition of chloramphenicol. The sensor showed a detection limit of 1.18 × 10^−13^ mol L^−1^, and a linear range of 5 × 10^−13^ to 4 × 10^−10^ mol L^−1^. Each sensor component offered improved sensitivity and selectivity features even when using complex matrix samples, essential to tracing antibiotic residues in food safety control [91].

The possibility of having a sensitive fluorescence system for selective detection of the mycotoxin patulin motivated the development of a system based on Ag NP/flake-like Zn-based MOF nanocomposite (AgNPs@ZnMOF) as an efficient support for MIP [92]. The Ag NPs were created inside the nano-pores of flake-like Zn MOF and the peroxidase-like activity of Ag NPs was greatly improved by the high surface area of MOF while the MIP for patulin ensured the selectivity. The fluorescence intensity, resulting from the product of catalyzed H_2_O_2_-terephthalic acid reaction, decreased linearly with increasing concentrations of patulin in a range of 0.1–10 µmol L^−1^ and a detection limit of 0.06 µmol L^−1^ [92].

In bioanalytical chemistry, the use of immunoassays is widespread, and the ELISA is considered the gold standard in many applications, owing to the highly sensitive detection [93]. Nonetheless, the research on biomimetic alternatives is very attractive, namely, for reducing production costs and enhancing the reagent stability. Innovation at this level can occur both by using MIPs, also known as synthetic antibodies, and by employing nanozymes as catalytic labels. This biomimetic approach has been proposed for detecting an organophosphate pesticide [93]. In this work, the 96-well array format was accomplished with the synthesis of MIP microspheres by precipitation polymerization, followed by immobilization on the plate by “grafting to” method, assisted by ionic liquid as binder [93]. The nanozyme label relied on the peroxidase-like activity of Pt NPs and was prepared in two steps. First, BSA-hapten conjugates were synthesized and the final Pt@BSA-hapten probe was obtained by mixing with a colloidal solution of Pt NPs. Colorimetric and surface-enhanced Raman scattering were used for signal detection because the substrate TMB was oxidized to a blue TMB^2+^, which also possesses a Raman signal. The proposed method showed a limit of detection of 1 ng mL^−1^ for triazophos [93]. Various biomimetic alternatives to common ELISA assays have been proposed. The detection of sulfadiazine, an antibacterial compound whose residues in food and environment from misuse are of health concern, was studied using Au@SiO_2_ and Au@Pt@SiO_2_ NPs [94,95]. The nanocomposites as labelling markers had the intrinsic peroxidase-like activity of Au NPs or Au@Pt bimetallic materials, and the SiO_2_ NPs offered large surface area and ease of surface functionalization. This ideal combination was further improved by preparing MIP films for selective recognition and reusability, allowing to develop assays whose results were comparable to those obtained by HPLC [94,95]. Other configurations have been proposed, such as the use of Pt@SiO_2_ NPs and a MIP for highly sensitive and selective detection of histamine with a limit of detection of 0.128 mg L^−1^ [96].

**Figure 11:**
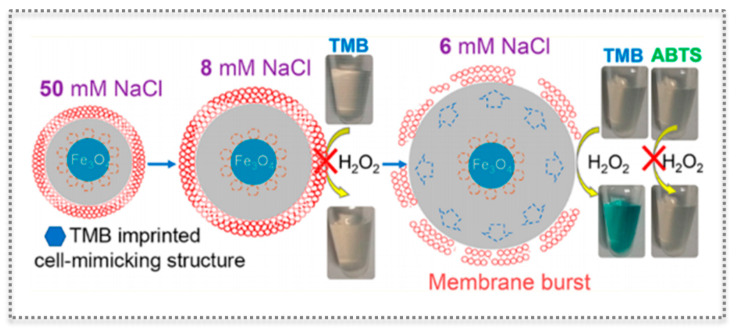
Cell-mimicking protocell of three components with a peroxidase-like iron oxide core as nucleus, a molecularly imprinted hydrogel shell as cytoplasm, and a lipid bilayer for biomedical applications. (Reproduced with permission [97], Copyright 2017, American Chemical Society).

The multiplicity of sensor design has been expanded by the report of a three-component functional cell-mimicking structure bearing a nuclear Fe_3_O_4_ peroxidase-like activity, a shell layer of a stimuli-responsive molecularly imprinted hydrogel representing the cytoplasm, and a lipid bilayer membrane [97] (Figure 11). Each component of the tripartite system had unique functions to deploy an interesting switch-like colorimetric system. The core Fe_3_O_4_ ensures the oxidation of the chromogenic substrate TMB (and similar, like ABTS) in the presence of H_2_O_2_. In turn, the MIP layer selectively recognizes TMB and is simultaneously sensitive to salt concentration by swelling or shrinking [97]. Finally, the elasticity of the lipid membrane keeps up with the swelling of the anionic MIP gel when lowering the salt concentration, until there is no more tolerance to volume change and the lipid layer bursts. Thus, the gradual “analog” gel volume change was reflected in a “digital” colorimetric output because the burst of the membrane allows access to TMB that is oxidized to produce color. Interestingly, controlled access to TMB can also be achieved by using melittin, a membrane-perturbing amphipathic peptide, which introduces channels in the membrane. The selectivity is ensured by the imprinted gel, while this approach enlarges the potential applications of the system [97].

## 5. Conclusions and Future Perspectives

**Figure 12:**
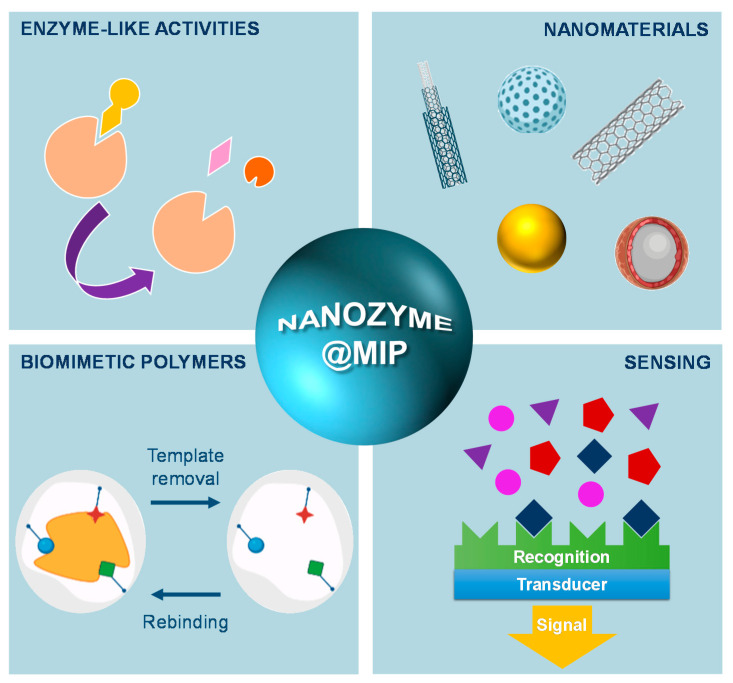
Schematic representation of the nanozyme action in combination with MIP materials.

In the quest for cost-effective and robust mimics of natural enzymes, intense research efforts have achieved progress in improving the selectivity and catalytic efficiency of artificial enzymes, by gathering a wide range of materials and approaches (Figure 12). Nanozymes have emerged as the next generation of enzyme mimics to overcome the existing challenges and broaden the application of synthetic catalysts. Despite the demonstrated advantages, the lack of specificity has always been a problem that hindered a faster and broader application of nanozymes. The synergy gained by using molecular imprinting technology to create selective substrate binding sites on nanozymes has been reasoned as a solution to solve the obstacle of missing specificity. Simultaneously, MIPs are also biomimetic, low-cost, and stable materials that can be produced on a large scale. The intrinsic catalytic activity of nanozymes can be improved by designing nanomaterial cores and proper surface functional groups, also benefiting from nanocomposites and doped nanomaterials. Concurrently, this diversity in composition, sizes, shapes, and surface properties calls for rigorous characterization and standardization of activity if practical implementation is foreseen. MIP technology is also resourceful, contributing with the possibility to obtain on-demand tailorable functional moieties and stimuli-responsive nanostructures. This biomimetic convergence is a hot research topic and new insights into functional engineered nanozymes@MIPs in the development of biosensing devices are expected to find auspicious biosensing applications soon. 

Once they have surpassed the current limitations and achieved the enhanced catalytic activity and specificity, imprinted nanozymes can be incorporated in portable, low-cost, and time-saving assays for novel point-of-care applications. These point-of-care applications linked to low-cost devices that rely on synthetic materials are a huge opportunity for developing new tools that allow global efforts and alliances in combating the current and future pandemics, as well as screening for chronic diseases, allowing earlier detection and earlier medical action, to improve treatment outcomes.

## Data Availability

Not applicable.
